# Prevalence of Gestational Diabetes Mellitus in urban and rural Tamil Nadu using IADPSG and WHO 1999 criteria (WINGS 6)

**DOI:** 10.1186/s40842-016-0028-6

**Published:** 2016-04-05

**Authors:** Balaji Bhavadharini, Manni Mohanraj Mahalakshmi, Ranjit Mohan Anjana, Kumar Maheswari, Ram Uma, Mohan Deepa, Ranjit Unnikrishnan, Harish Ranjani, Sonak D Pastakia, Arivudainambi Kayal, Lyudmil Ninov, Belma Malanda, Anne Belton, Viswanathan Mohan

**Affiliations:** 1 Madras Diabetes Research Foundation, 4, Conran Smith Road, Gopalapuram, Chennai, 600 086 India; 2 Seethapathy Clinic and Hospital, Chennai, India; 3 College of Pharmacy, Purdue University, West Lafayette, IN USA; 4 International Diabetes Federation, Brussels, Belgium

**Keywords:** Gestational diabetes mellitus, IADPSG criteria, WHO 1999 criteria, Prevalence, Asian Indians, South Asians

## Abstract

**Background:**

To determine the prevalence of Gestational Diabetes Mellitus (GDM) in urban and rural Tamil Nadu in southern India, using the International Association of Diabetes and Pregnancy Study Groups (IADPSG) and the World Health Organization (WHO) 1999 criteria for GDM.

**Methods:**

A total of 2121 pregnant women were screened for GDM from antenatal clinics in government primary health centres of Kancheepuram district (*n* = 520) and private maternity centres in Chennai city in Tamil Nadu (*n* = 1601) between January 2013 to December 2014. Oral glucose tolerance tests (OGTT) were done after an overnight fast of at least 8 h, using a 75 g glucose load and venous samples were drawn at 0, 1 and 2 h. GDM was diagnosed using both the IADPSG criteria as well as the WHO 1999 criteria for GDM.

**Results:**

The overall prevalence of GDM after adjusting for age, BMI, family history of diabetes and previous history of GDM was 18.5 % by IADPSG criteria with no significant urban/rural differences (urban 19.8 % vs rural 16.1 %, *p* = 0.46). Using the WHO 1999 criteria, the overall adjusted prevalence of GDM was 14.6 % again with no significant urban/rural differences (urban 15.9 % vs rural 8.9 %, *p* = 0.13).

**Conclusion:**

The prevalence of GDM by IADPSG was high both using IADPSG as well as WHO 1999 criteria with no significant urban/rural differences. This emphasizes the need for increasing awareness about GDM and for prevention of GDM in developing countries like India.

**Electronic supplementary material:**

The online version of this article (doi:10.1186/s40842-016-0028-6) contains supplementary material, which is available to authorized users.

## Background

The prevalence of diabetes mellitus (DM) is increasing worldwide and more so in developing countries such as India [1, 2]. Along with the rising tide of the current epidemic of diabetes, the prevalence of gestational diabetes mellitus (GDM), defined as any degree of glucose intolerance with onset or first recognition during pregnancy, is also on the rise [3, 4]. GDM increases the risk of complications in both the mother and child and early detection and management improves outcomes for both [5, 6]. In 2013, 6 million women in India had some form of hyperglycemia in pregnancy, of which 90 % were GDM [6]. Racial/ethnic differences in the prevalence of GDM have been documented, with a higher prevalence among Native American, Asian, African-American, and Hispanic populations compared to non-Hispanic Whites [7]. GDM is usually asymptomatic and is most commonly diagnosed by routine screening during pregnancy. Unfortunately, there is little agreement on the best screening and diagnostic tests for GDM. The International Association of Diabetes and Pregnancy Study Group (IADPSG) criteria was introduced in the year 2010 and it has found fairly wide acceptance [8]. However, there have been some reports that it may lead to inflated prevalence rates of GDM [1, 8–10]. In this paper, we report on the prevalence of GDM in urban and rural Tamil Nadu in southern India using the IADPSG criteria and compare the same with prevalence rates obtained using the World Health Organization (WHO) 1999 criteria for GDM.

## Methods

This study is part of the Women in India with GDM Strategy (WINGS) project of the International Diabetes Federation carried out in Chennai city (urban) and rural antenatal clinics in Tamil Nadu in south India. The study was conducted between January 2013 and December 2014. Consecutive pregnant women were screened at their first booking at 15 government primary health centres in Kancheepuram district and 6 private health centres at Chennai city in Tamil Nadu state. Written informed consent was obtained in the local language from all participants and the study was approved by the Institutional Ethics Committee of the Madras Diabetes Research Foundation (MDRF). All procedures followed were in accordance with the ethical standards and in keeping with the Declaration of Helsinki 1975, as revised in 2008. Permission was also obtained from the Directorate of Public Health and the Ministry of Health, Government of Tamil Nadu to conduct the study in the primary health centres. Clinical information including obstetric history, family history of diabetes as well as current and past medications was collected using a structured questionnaire.

Height was measured using a stadiometer (SECA Model 213, Seca Gmbh Co, Hamburg, Germany) to the nearest 0.1 cm and weight was measured with an electronic weighing machine (SECA Model 803, Seca Gmbh Co) to the nearest 0.1 kilogram. The body mass index (BMI) was calculated as weight (kg) divided by height (in metres) squared. Participants were requested to report in the fasting state (at least 8 h of overnight fasting), between 7 and 9 am on the day of blood collection. A fasting venous sample was drawn for plasma glucose estimations. 82.5 g of anhydrous glucose (equivalent to 75 g of monohydrate glucose) was then dissolved in 300 ml of water and was given to the pregnant women who consumed it within 5 min. Further venous samples were drawn at 1 h and 2 h after the ingestion of oral glucose.

Plasma glucose (PG) was estimated by the glucose oxidase–peroxidase method using autoanalyser AU2700 (Beckman, Fullerton, CA). Glycated haemoglobin (HbA1c) was measured using high performance liquid chromatography (HPLC) using Variant machine (BIORAD, Hercules, CA). The intra and inter-assay coefficients of variation (CV) for the glucose and HbA1c ranged from 0.78–1.68 % and 0.59–1.97 % respectively. All samples were processed in our laboratory which is certified by the College of American Pathologists (CAP) and by the National Accreditation Board for Testing and Calibration Laboratories (NABL), Government of India.

### Definitions

GDM was diagnosed by IADPSG criteria, if any one of the fasting, 1 h or 2 h PG values met or exceeded 5.1 mmol/L (≥92 mg/dl), 10.0 mmol/L (≥180 mg/dl) and 8.5 mmol/L (≥153 mg/dl) [11] respectively. As per the IADPSG criteria, in the first trimester, GDM was diagnosed using only the fasting glucose estimations, while in 2nd/3rd trimester, GDM was diagnosed using an oral glucose tolerance test (OGTT).

The World Health Organization (WHO) 1999 criteria [12], which diagnoses GDM using 2 h PG value of 7.7 mmol/l (≥140 mg/dl) was applied to the results, to compare the prevalence rates with those obtained using the IADPSG criteria.

### Statistical analysis

All analyses was done using Windows based SPSS statistical package (version 15.0, Chicago, IL). Estimates were expressed as mean ± standard deviation or proportions. To compare continuous variables, *t* tests were used while chi square tests were used to test differences in proportions. *P*-value <0.05 was considered significant. A multivariable logistic regression model was developed to identify factors associated with gestational diabetes using GDM diagnosis according to IADSPG criteria as the dependent variable and independent variables were chosen based on p value <0.2 in univariate analysis or were clinically relevant.

## Results

**Fig. 1:**
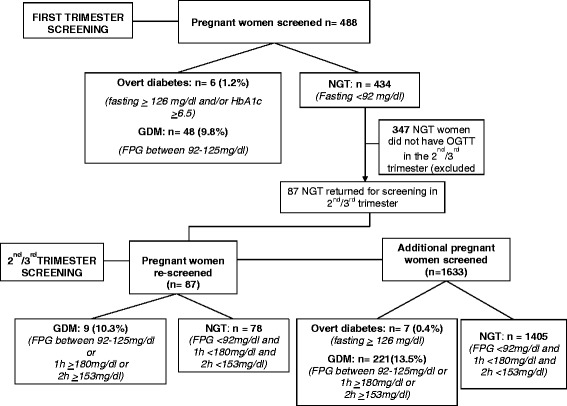
Schedule of the screening done in the first and 2^nd^/3^rd^ trimester in this study

A total of 2507 consecutive pregnant women were approached to participate in the WINGS screening programme of whom 2121 consented (84.6 %) which included 520 from rural, and 1601 from urban, centres. As shown in Fig. 1, a total of 488 women underwent screening in the first trimester. GDM was diagnosed in 48 women (9.8 %) using the IADPSG criteria while 6 (1.2 %) had overt diabetes, i.e., fasting PG ≥ 7 mmol/l (≥126 mg/dl) and/or HbA1c ≥6.5 %. As part of WINGS protocol, in the pilot phase, we did not expect women to return in the 2^nd^/3^rd^ trimester for repeat OGTT unlike in the Model of Care phase of WINGS where we followed women right through the pregnancy. Nevertheless, of the remaining 434 women, 87 who screened negative in the first trimester, returned for repeat OGTT in the 2^nd^/3^rd^ trimester. The rest (*n* = 347) who did not return for a repeat OGTT in their 2^nd^/3^rd^ trimester, were excluded from further analysis.

In the 2^nd^/3^rd^ trimester, 1633 women who were not screened in the first trimester were screened using the IADPSG criteria. GDM was diagnosed in 221 (13.5 %) women while 7 (0.4 %) women had overt diabetes. Among the 87 women who had normal glucose tolerance (NGT) in the first trimester screening, 9 (10.3 %) developed GDM in the second/third trimester.

In both urban and rural populations, prevalence of GDM was significantly higher by the IADPSG criteria when compared to the WHO 1999 criteria. Table 1 shows that the overall prevalence (unadjusted) of GDM by the IADPSG criteria was 15.7 % (*n* = 278), while in urban areas, it was 16.1 % and in rural areas, 14.4 % (*p* = 0.37). After adjusting for age, BMI, family history of diabetes and previous history of GDM, the overall prevalence by IADPSG criteria was 18.5 % (urban 19.8 % vs rural 16.1 %, *p* = 0.46).

**Table 1:** Prevalence of gestational diabetes based on IADPSG and WHO 1999 criteria

Criteria	Overall prevalence (*n* = 1774)	Urban (*n* = 1301)	Rural (*n* = 473)
Unadjusted prevalence rates			
IADPSG Criteria	278 (15.7 %)	210 (16.1 %)	68 (14.4 %)
WHO 1999 criteria	186 (10.5 %)	161 (12.4 %)	25 (5.3 %)
Adjusted prevalence rates^a^			
IADPSG Criteria	18.5 %	19.8 %	16.1 %
WHO 1999 criteria	14.6 %	15.9 %	8.9 %

^a^Adjusted for age, BMI, family history of diabetes and previous history of GDM

If the WHO 1999 criteria was used, the unadjusted overall prevalence of GDM was 10.5 % (*n* = 186) [urban 12.4 % (*n* = 161) vs rural 5.3 % (*n* = 25), p < 0.001]. However, after adjusting for age, BMI, family history of diabetes and previous history of GDM, the urban/rural differences disappeared using the WHO 1999 criteria also (urban 15.9 % vs rural 8.9 %, *p* = 0.13).

**Fig. 2:**
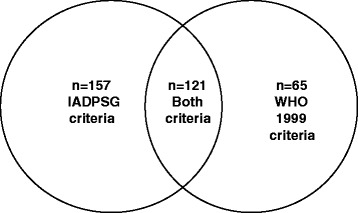
Venn diagram depicting the GDM identified by both criteria

Of the 278 women identified by IADPSG criteria, 121 (43.5 %) were picked up by the WHO 1999 criteria. Conversely, of the 186 women identified by the WHO 1999 criteria, IADPSG picked up 121 (65.1 %) of GDM (Fig. 2). Thus, 121 pregnant women were diagnosed by both IADPSG and WHO 1999 criteria (agreement, kappa = 0.45).

Table 2 shows the general characteristics of the 1774 study subjects in urban and rural areas. Women with GDM in urban areas were significantly older, had higher BMI and lower levels of fasting and HbA1c (*p* < 0.001) compared to those in rural areas.

**Table 2:** General characteristics of the GDM and non GDM diagnosed by IADPSG criteria in urban and rural areas

Parameter	URBAN (*n* = 1301)			RURAL (*n* = 473)			*p* value*	OVERALL (*N* = 1774)			
	Overall	GDM** (*n* = 210)	NON GDM (*n* = 1091)	Overall	GDM** (*n* = 68)	NON GDM (*n* = 405)		Overall	GDM (*n* = 278)	NON GDM (*n* = 1496)	*p* value***
Age	26.2 ± 4.1	27.4 ± 4.3	25.9 ± 4.0	24 ± 2.9	23.9 ± 2.5	23.9 ± 3.0	<0.001	25.6 ± 3.9	26.5 ± 4.2	25.4 ± 3.8	0.0238
BMI	24.7 ± 4.9	26.2 ± 4.5	24.6 ± 4.9	22.1 ± 3.7	22.6 ± 3.9	22.1 ± 3.6	<0.001	24.2 ± 4.7	25.4 ± 4.6	24.0 ± 4.7	<0.001
Family history of T2DM	366 (28.1 %)	82 (39 %)	284 (26 %)	51 (10.8 %)	12 (17.6 %)	39 (9.6 %)	<0.001	417 (23.5 %)	94 (33.8 %)	323 (21.6 %)	0.0001
Previous history of GDM	25 (1.9 %)	12 (5.7 %)	13 (1.2 %)	1 (0.2 %)	0	1 (0.2 %)	<0.001	26 (1.5 %)	12 (4.3 %)	14 (0.9 %)	0.0062
Fasting (mg/dl)	80 ± 12.8	93 ± 10.4	77 ± 11.6	81 ± 12.4	96 ± 11.3	78 ± 10.5	0.1355	80.6 ± 12.6	94.1 ± 10.7	78.0 ± 11.3	<0.001
HbA1c (%)	4.9 ± 0.6	5.1 ± 0.5	4.8 ± 0.5	5.1 ± 0.6	5.3 ± 0.6	5.0 ± 0.4	<0.001	4.9 ± 0.53	5.1 ± 0.4	4.9 ± 0.5	<0.001

*p value comparing overall urban vs rural

**p value significant (<0.005), comparing GDM urban vs GDM rural

***p value comparing overall “GDM vs non GDM”

Multivariable logistic regression models were used to identify factors associated with gestational diabetes based on the IADPSG criteria (Table 3). The variables that had a *p* value <0.2 in univariate analysis or were clinically relevant were used in the multiple logistic regression. HbA1c, previous history of GDM, family history of diabetes and age were significantly associated with GDM in this model.

**Table 3:** Multivariable logistic regression showing factors independently associated with gestational diabetes mellitus diagnosed by IADPSG criteria

Variable	Odds ratio (95 % CI), *p* value
HbA1c (at booking)	2.91 (1.69–3.12), *p* < 0.001
Previous history of GDM	3.63 (1.48–8.90), *p* = 0.005
Family history of diabetes	1.54 (1.11–2.15), *p* = 0.009
Age (at booking)	1.03 (1.00–1.08), *p* = 0.05
Body mass index (at booking)	1.02 (0.99–1.05), *p* = 0.14
Parity^a^	1.00 (0.74–1.35), *p* = 0.99

^a^ *Primi mothers as reference*

## Discussion

The study reports the following findings:

1. The prevalence of GDM based on the IADPSG criteria, after adjusting for age, BMI, family history of diabetes and previous history of GDM was 18.5 % and based on the WHO 1999 criteria, it was 14.6 %.
2. The prevalence rates of GDM were not significantly different between urban and rural areas both using the IADPSG criteria and WHO 1999 criteria after correcting for the confounders.
3. In the multivariable logistic regression, HbA1c, previous history of GDM, family history of diabetes and age were significantly associated with GDM.

Several criteria for diagnosing GDM have been recommended by various national and international bodies including the American Diabetes Association (ADA), Australasian Diabetes in Pregnancy Study Group (ADIPS), Canadian Diabetes Association (CDA), European Association for the study of Diabetes (EASD), International Association of the Diabetes and Pregnancy Study Groups (IADPSG), International Classification of Diseases (ICD), National Diabetes Data Group (NDDG), the World Health Organization (WHO) and the Diabetes In India Pregnancy Study Group of India (DIPSI). These criteria differ in their requirement for the subject to be in a fasting state, the number of samples needed, the amount of glucose administered and blood glucose thresholds for GDM detection [13]. Not surprisingly, the prevalence rates of GDM also vary according to the criteria used. In this paper, we report on the prevalence of GDM by the IADPSG and the WHO 1999 criteria.

Comparing the prevalence rates with other GDM prevalence studies carried out globally using the IADPSG criteria, a prevalence of 8.9 % has been reported in Sri Lanka [14] and 2.6 % in Thailand [15] and between 2–6 % in Europe [16]. Using the WHO 1999 criteria, a prevalence of 7.2 % was reported in Sri Lanka [15], 9.7 % in Bangladesh [17], 11.4 % in Malaysia [18], 20.6 % in United Arab Emirates [19] and 16.3 % in Qatar [20]. Table 4 summarizes the prevalence of GDM reported in some of the recent studies conducted worldwide [21–28].

**Table 4:** Studies on prevalence of gestational diabetes mellitus–worldwide

Author Name	City/Country	Sample Size	Prevalence	Criteria used for GDM diagnosis
Agarwal et al. (2007)	Al Ain, United Arab Emirates	1172	20.6 %	WHO 1999
Tan et al. (2007)	Malaysia	1600	11.4 %	WHO 1999
Bener et al. (2011)	Doha, Qatar	1608	16.3 %	WHO 1999
Moses et al. (2011)	Australia	1275	9.6 % 13.0 %	ADIPS IADPSG
Dahanayaka et al. (2012)	Sri Lanka	405	8.9 %	IADPSG
Jenum et al. (2012)	Oslo, Norway	823	13 % 31.5 %	WHO 1999 IADPSG
Kalter-Leibovivi et al. (2012)	Israel	3,345	9 %	IADPSG
Reyes-Munoz E et al. (2012)	Mexico	803	10.3 % 30.1 %	ADA 2005 IADPSG
Kanjana et al. (2013)	Thailand	6324	2.6 %	IADPSG
Duran et al. (2014)	Madrid, Spain	1750 1526	10.6 % 35.5 %	Carpenter & Coustan IADPSG
Shang et al. (2014)	China	3083	19.9 % 7.98 %	IADPSG ADA 2005
Liao et al. (2014)	Chengdu, China	5630	11.7 % 24.5 %	ADA 2005 IADPSG
Leng et al. (2015)	Tianjin, China	18589	8.1 % 9.3 %	WHO 1999 IADPSG
Hung et al. (2015)	Taoyuan, China	3056 3641	4.6 % 12.4 %	ADA 2005 IADPSG
Sibartie et al. (2015)	Australia	10103	3.4 % 3.5 %	ADIPS IADPSG
Ethridge et al. (2015)	Ohio, United States of America	8390	4 % 3.3 %	Carpenter & Coustan IADPSG
O’Sullivan et al. (2016)	Galway, Ireland	5500	12.4 % 9.4 %	IADPSG WHO 1999

**Fig. 3:**
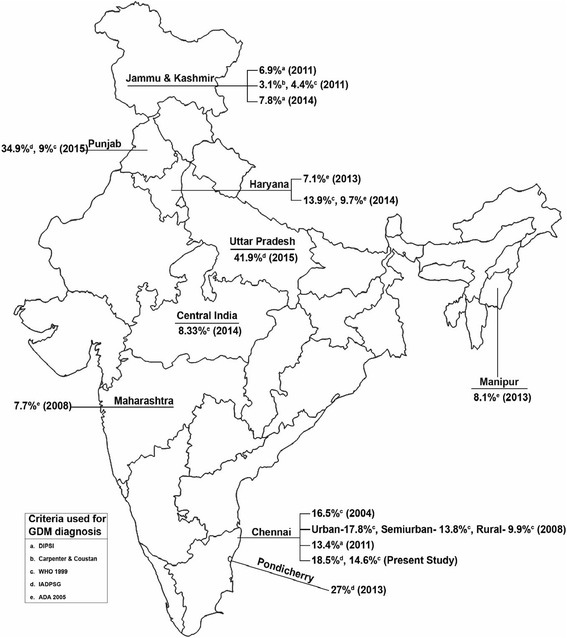
Graphical representation of GDM prevalence across India

Table 5 and Fig. 3 presents a review of various studies on GDM prevalence carried out in India since 2004. Using the Diabetes in Pregnancy Study Group of India (DIPSI) criteria, which diagnoses GDM based on a non fasting 2 h OGTT, a prevalence of 6.9 % was reported in Jammu [29]. Using the ADA criteria, which recommends a two step procedure, i.e., a 50 g glucose challenge test followed by 100 g confirmatory OGTT, a prevalence of 7.1 % was reported in Haryana [30], 7.7 % in Maharashtra [9] and 8.1 % in Manipur [31] and 3.1 % in Kashmir [32]. Using the IADPSG criteria, a high prevalence (27 %) was reported in Puducherry [10]. Using WHO 1999 criteria, a prevalence of 16.5 % was reported in an earlier study carried out in Chennai [33], and a prevalence of 4.4 % was reported in Kashmir [32]. Recent studies from India by Arora et al. [34] have also reported higher prevalence rates of GDM (34.9 %) using the IADPSG criteria. Though their sample size was large, the authors had used 2 h capillary measurements instead of venous plasma samples albeit with adjustment for the values. This might explain, at least partly, the differences from our study.

**Table 5:** Studies on prevalence of gestational diabetes mellitus in India

Author Name	City/State	Sample Size	Prevalence	Criteria used for GDM diagnosis
Seshiah et al. (2004)	Government Maternity Hospital, Chennai	3674	16.5 %	WHO 1999
Seshiah et al. (2008)	Chennai, South India	4151	Urban-17.8 % Semiurban-13.8 % Rural-9.9 %	WHO 1999
Swami et al. (2008)	Tertiary care hospital in Maharashtra	1225	7.7 %	ADA 2005
Seshiah et al. (2011)	Chennai, South India	1463	13.4 %	DIPSI
Wahi et al. (2011)	Govt Medical College Hospital, Jammu region	2025	6.9 %	DIPSI
Nayak et al. (2013)	Pondicherry Institute of Medical Science	304	27 %	IADPSG
Vanlalhruaii et al. (2013)	Regional Institute of Medical Sciences Manipur	300	8.1 %	ADA 2005
Rajput et al. (2013)	Post Graduate Institute of Medical Sciences Haryana	607	7.1 %	ADA 2005
Zargar et al. (2004)	Sher-i-Kashmir Institute of Medical Sciences	2000	3.1 % 4.4 %	Carpenter & Coustan WHO 1999
Raja et al. (2014)	Government Medical College Srinagar Kashmir valley	306	7.8 %	DIPSI
Rajput et al. (2014)	Rural Haryana	900	13.9 % 9.7 %	WHO 1999 ADA 2005
Kalyani et al. (2014)	Central India	300	8.33 %	WHO 1999
Arora et al. (2015)	Ludhiana, Punjab	5100	34.9 % 9 %	IADPSG WHO 1999
Gopalakrishnan V et al. (2015)	Sanjay Gandhi Postgraduate Institute of Medical Sciences, Lucknow, Uttar Pradesh, India.	332	41.9 %	IADPSG
Present study	Chennai, India	1774	18.5 % 14.6 %	IADPSG WHO 1999

The prevalence of GDM in our study was 26.7 % higher by the IADPSG criteria compared to the WHO 1999 criteria. This is similar to the 25 % higher prevalence reported by O’Sullivan et al. [35] [IADPSG–12.4 % vs. WHO–9.4 %]. In another study in Sri Lanka, the prevalence of GDM was 23.6 % higher using IADPSG compared to WHO 1999 criteria [15]. Studies from China showed the prevalence to be higher by IADPSG (19.9 %) when compared to ADA criteria (7.9 %) [36]. A study from Taiwan [37] showed that the IADPSG criteria increased the prevalence of GDM from 4.6 % (by ADA criteria) to 12.4 %. A study from Canada reported an increase in the rates of GDM from 7.9 % using ADA criteria to 9.4 % if IADPSG criteria were used [38]. In Spain, applying the IADPSG criteria was associated with a 3.5-fold increase in GDM prevalence [39]. The higher percentage increase could perhaps to attributed to ethnic differences in fasting hyperglycemia. Earlier studies have shown that Asian Indians have higher fasting hyperglycemia compared to Caucasians [40, 41]. Gopalakrishnan et al. [42] reported 41.9 % prevalence of GDM in their study, of whom 70.5 % had abnormal fasting blood glucose alone. Nayak et al. [43] also showed that 63.8 % of GDM identified by the IADPSG criteria had fasting hyperglycemia. Another study by Moradi et al. [44] showed that 48 % of GDM identified by IADPSG had elevated fasting blood glucose levels alone. Results from our study shows that even among Asian Indians, rural women with GDM have higher fasting hyperglycemia. It is interesting to note, that although these women are significantly younger, less heavy, have less family history of diabetes, their fasting plasma glucose levels and HbA1c are higher compared to women with GDM in urban area. This could account for the discrepancy between the prevalence rates by IADPSG and WHO 1999 criteria in the rural population. Given that the two criteria identifies different sets of patients, omitting the fasting criteria (as in the WHO 1999 criteria) would tend to miss a lot of GDM cases especially in the rural population.

It has also been shown that fasting hyperglycemia by IADPSG criteria is associated with increased perinatal complications [45]. The arguments in favor of using IADPSG criteria are therefore based on pregnancy outcomes, and early screening and diagnosis of GDM helps to initiate treatment earlier (usually medical nutrition therapy) [46, 47]. Thus, whereas on one hand, there is indeed an increase in prevalence of GDM, on the other hand, identifying more women and starting lifestyle changes promises better outcomes [39].

A Sri Lankan study reported 51.1 % agreement between IADPSG and WHO 1999 criteria [15] which is similar to the present study, where 45 % agreement was noted.

We have earlier reported on the necessity for doing fasting OGTTs for diagnosing GDM [48] and also on the need for doing venous plasma samples [49]. In this paper we report on fasting OGTTs using venous plasma samples using both the IADPSG and WHO 1999 criteria.

Earlier studies have shown that advanced maternal age, obesity, and family history of diabetes to be associated with GDM [3]. These findings are consistent with the present study findings, which reveals that, HbA1c, previous history of GDM, family history of diabetes and age were found to be associated with GDM diagnosed using the IADPSG criteria.

This study has several strengths: (i) both urban and rural areas were sampled; (ii) large sample size; (iii) this is one of the first studies from India to report on the differences in prevalence of GDM by both IADPSG and WHO 1999 criteria in urban/rural areas (iv) screening was done during the first trimester and in the 2^nd^/3^rd^ trimester using the IADPSG criteria. Traditionally, screening for GDM is delayed until 2nd or early 3rd trimester since the diabetogenic effects of pregnancy increases with gestational age, and delayed testing would maximize the detection rate [50, 51]. However, early identification gives time for appropriate intervention which could help reduce complications. Moreover, as shown in our study, screening in the first trimester also provides an opportunity to detect previously undiagnosed overt diabetes as well as GDM. In our study, first trimester screening identified 1.2 % of overt diabetes and 9.8 % GDM. A study from Trichy [52] recently reported GDM prevalence of 13.9 % in first trimester. Data from Oklahoma shows that among American Indians, prevalence of GDM and overt diabetes in first trimester was 24 % and 0.4 % respectively [53]. There is insufficient data from India on the prevalence of overt diabetes in the first trimester and hence the findings from this study are significant. As per the recommendations of IADPSG criteria, women who are labeled as having normal glucose tolerance in the first trimester should undergo a repeat OGTT in the 2^nd^/3^rd^ trimester. However, in our study, only 87 women of the 434 returned for repeat OGTT which is one of the limitations of this study. A recent study by Morikawa et al. [54] from Japan, showed that women who were diagnosed as having normal glucose tolerance (NGT) in the first trimester remained as NGT throughout their pregnancy, despite the significant increase in insulin resistance. In contrast, results from our study shows that among those who returned for a repeat screening (*n* = 87), 10.3 % developed GDM. Similar findings emphasizing the need for repeat screening have been reported earlier in Hungarian women, where the GDM prevalence was noted to increase with advancing gestation [55]. This therefore highlights the importance of repeat screening among women who screened negative in the first trimester in populations like ours which have a higher risk for GDM. Another limitation of the study is that, pregnant women included in the study were from a few selected antenatal clinics in urban and rural areas in Tamil Nadu and hence the results may not be representative of the GDM rates in the country as a whole. Finally, there were some significant differences between the 1774 women who participated and the 386 women who refused to participate, which is yet another limitation (Additional file: 1 Table S1).

## Conclusions

The prevalence of GDM in Tamil Nadu was found to be 15.7 % (adjusted 18.5 %) by IADPSG criteria and 10.5 % (adjusted 14.6 %) using the WHO 1999 criteria. There were no urban rural differences using both criteria suggesting that the rural areas in southern India are also fasting catching up with reference to rising GDM prevalence rates. This emphasizes the need for increasing awareness about GDM and taking steps to prevent GDM in India and other developing countries.

### Ethical standard

This study was approved by the Institutional Ethics Committee of the Madras Diabetes Research Foundation, Chennai, India [Dated 7th November 2012].

### Human and animal rights disclosure

All human rights were observed in keeping with Declaration of Helsinki 2008 (ICH GCP) and the Indian Council of Medical Research (ICMR) guidelines. There are no animal rights issues in this study.

### Informed consent disclosure

Written informed consent was obtained from all participants before being included in the study.

## Additional file

Additional file 1: Table S1. Difference between those who participated and those who refused to participate. (DOC 33 kb)
