# Supplementary material for: Prevalence of Gestational Diabetes Mellitus in urban and rural Tamil Nadu using IADPSG and WHO 1999 criteria (WINGS 6)
Source: Clin Diabetes Endocrinol. 2016 Apr 5;2:8. doi: 10.1186/s40842-016-0028-6 (PMC5471800; doi:10.1186/s40842-016-0028-6)
Supplement: Additional file 1: Table S1. — Difference between those who participated and those who refused to participate. (DOC 33 kb) [file 40842_2016_28_MOESM1_ESM.doc]

**Additional file 1: Table S1. Difference between those who participated and those who refused to participate**

| **SNO** | **PARAMETER** | **THOSE WHO PARTICPATED (n=1774)** | **THOSE WHO REFUSED TO PARTICIPATE (n=386)** | **p VALUE** |
| --- | --- | --- | --- | --- |
| 1 | Age (in years) | 25.6 ± 3.9 | 23.9 ± 3.4 | <0.001 |
| 2 | Gestational weeks at screening (weeks) | 21.9 ± 7.0 | 24.5 ± 8.6 | <0.001 |
| 3 | BMI (kg/m^2^) | 24.3 ± 4.7 | 23.1 ± 4.7 | 0.0001 |
| 4 | Previous history of GDM (%) | 26 (1.5%) | 0 | - |
| 5 | Family history of diabetes (%) | 417 (23.5%) | 46 (11.9%) | <0.001 |
| 6 | Residence (%)  Urban  Rural | 1301 (73.3%)  473 (26.7%) | 222 (57.5%)  164 (42.5%) | <0.001  <0.001 |
| 7 | Primi mothers (%) | 744 (42.1%) | 172 (44.6%) | 0.4149 |
